# Malignant Endocarditis

**Published:** 1902-04-19

**Authors:** 


### MALIGNANT ENDOCARDITIS.

Of late years it has been generally considered that malignant endocarditis, even though it may occur in association with rheumatic fever, is due to the implantation of some foreign septic or pathogenic micro-organism, a pus-producing organism, a pneumococcus, or even a bacillus typhosus. At the last meeting of the Royal Medical and Chirurgical Society, however, a paper read by Dr. Poynton and Dr. Alexander Paine raised the question whether certain cases of malignant endocarditis are not, after all, rheumatic. The relation between malignant endocarditis and rheumatic fever is, they consider, a very close one, especially in regard to certain cases of malignant endocarditis with a previous history of rheumatic fever and in the course of which rheumatic manifestations are apt to occur. The theory that rheumatic fever is itself a microbic disease materially alters the problem. The evidence of the cause of rheumatic fever being a diplococcus is, they say, now extremely strong, and the question arises whether there is not a group of cases of malignant endocarditis which are rheumatic in origin. Indeed it would seem hard to deny to the rheumatic diplococcus, if there is such a creature, a privilege extended to so many other micro-organisms. The authors of the paper show that a diplococcus can be isolated from cases of malignant endocarditis which can be grown in pure culture, which will reproduce the disease in rabbits, and which resembles the diplococcus of rheumatic fever in its morphology and cultural characteristics. The diplococcus of rheumatic fever will produce malignant endocarditis in rabbits on the one hand, and the diplococcus from certain cases of malignant endocarditis will produce rheumatic fever in rabbits on the other. Finally, every grade of valvulitis, from simple valvulitis to malignant ulcerative endocarditis, can be produced from these two microbes, and the authors do not regard the distinctions which they find between these two diplococci as sufficient to constitute them separate and independent organisms. If all this be so, then we shall have to face the question why do certain cases become malignant? Hitherto, the answer has been that some malignant organism has obtained access to the body, but if the mischief though malignant be still rheumatic, many new problems must arise.

